# Acknowledgement to Reviewers 2024

**DOI:** 10.3389/ijph.2025.1608757

**Published:** 2025-06-25

**Authors:** IJPH Editorial Office

**Affiliations:** Swiss School of Public Health (SSPH+), Zürich, Switzerland

The Editors of the *International Journal of Public Health* would like to thank all Reviewers in 2024 for the time they have spent and the valuable contributions they have made to ensure the scientific quality of the journal.

Desta Erkalo Abame, Ethiopia

Charles Abongomera, Switzerland

Laura Acosta, Argentina

Adekunle Adedeji, Germany

Bruria Adini, Israel

Claudia Agnoli, Italy

Andreas A. Agudelo-Suárez, Colombia

Prince Agwu, Nigeria

Hamed Ahmadinia, Finland

Sedef Akgungor, Türkiye

Ali Akhtar, Pakistan

Akanni Akinyemi, Nigeria

Fatme Al Anouti, United Arab Emirates

Tunde Alabi, Nigeria

Violeta Alarcão, Portugal

Jesus Alejandro Aldana Lopez, Mexico

Mulubirhan Assefa Alemayohu, Italy

Shehzad Ali, Canada

Karim A. Mohamed Al-Jashamy, Malaysia

Saud Alsanad, Saudi Arabia

Anmar Al-Taie, Türkiye

Zeynep Meva Altaş, Türkiye

Henrique Telo Alves, Portugal

Rebecca Amati, Switzerland

Paulo Afonso André, Brazil

Italo F. Angelillo, Italy

Donato Angelino, Italy

Darijana Antonić, Bosnia and Herzegovina

Zeus Aranda, Mexico

Daniel Arnal, Spain

Marta Asensi, Italy

Rufin Kouassi Assare, Côte d’Ivoire

Aneta Atanasovska, North Macedonia

Muhammad Atiq, Pakistan

Yupin Aungsuroch, Thailand

Alba Ayala, Spain

Sina Azadnajafabad, United States

Irene Falgas Bague, Switzerland

Elhadj Balde, Luxembourg

Joseph Kimuli Balikuddembe, China

Delia Bancila, Denmark

Caroline Barakat, Canada

Josleen Barathie, Lebanon

Antonio Barbato, Italy

Marco Bardus, United Kingdom

Peter Michael Barrett, Ireland

Tibor Baska, Slovakia

Mattis Beckmannshagen, Germany

Martina Behanova, Austria

Mark Belger, United Kingdom

Sarni Maniar Berliana, Indonesia

Marlene Joannie Bewa, United States

Shiva Bhandari, United States

Emmanuel Bhaskar, India

Regien Biesma, Netherlands

Annibale Biggeri, Italy

Hashem Bishara, Israel

Bijit Biswas, India

Daniela Bobáková, Slovakia

Giovanna Boccuzzo, Italy

Lucia Bosakova, Slovakia

Charlotte Boven, Belgium

Jadranka Bozikov, Croatia

Elise Braekman, Belgium

Alexandra Brazinova, Slovakia

Sven Bremberg, Sweden

Karisa Brina, Brazil

Tristi Brownett, United Kingdom

Michał Brzeziński, Poland

John Bua, Uganda

Nolwenn Bühler, Switzerland

Michela Bulgari, Italy

Stefan Bushuven, Germany

Joanna Busza, United Kingdom

John Paul Byagamy, Uganda

Erwin Calgua, Guatemala

Luis Calmeiro, Singapore

Maureen Canavan, United States

Eva Cantoni, Switzerland

Nicola Caranci, Italy

Elsy Cárdenas-García, Mexico

Santiago Carranco, Ecuador

Rodrigo Casanueva, Chile

Raphaële Castagné, France

Dolores Catelan, Italy

Sabanur Çavdar, Türkiye

Monique Chaaya, Lebanon

Kenneth Chambaere, Belgium

Laurent Chambaud, France

Jiaying Chen, China

Tara Chen, Belgium

Mengling Cheng, Switzerland

Afona Chernet, Switzerland

Antonio Cherubini, Italy

Jyoti Chhibber-Goel, India

Paolo Chiodini, Italy

Marco Matteo Ciccone, Italy

Aaron Cohen, United States

Alessandra Costanza, Switzerland

Simona Costanzo, Italy

Sara Cottler-Casanova, Switzerland

Jean Tenena Coulibaly, Côte d’Ivoire

Gaoussou Coulibaly, Côte d’Ivoire

Lisa Cross, United States

Sarah Curtis, United Kingdom

Tracy Cushing, United States

Pedro Da Anunciação, Portugal

Giulia Dagliana, Italy

Margie Danchin, Australia

Walter De Caro, Italy

David DeGroot, United States

Hengameh R. Dehkordi, Brazil

Fernando Del Fiol, Brazil

Peter Delobelle, South Africa

Daniel Demant, Australia

Michael J. Deml, Switzerland

Giuseppe Di Martino, Italy

Zeno Di Valerio, Italy

Joseph Dieleman, United States

Pablo Villalobos Dintrans, Chile

Martin Dlouhy, Czechia

Ana Luiza Gomes Domingos, Brazil

Keren Dopelt, Israel

Selvira Draganovic, Bosnia and Herzegovina

Vadim Dukhanin, United States

Lionel Dumont, Switzerland

Camille Duveau, Belgium

Anna Maria Dzielska, Poland

Tamer Edirne, Türkiye

Piet van Eeuwijk, Switzerland

Zsuzsanna Elekes, Hungary

Thistle Elias, United States

Tara Elton-Marshall, Canada

Selahattin Tolga Er, Germany

Mehtap Eroglu, Türkiye

Ugochukwu Anthony Eze, Nigeria

Marta Fadda, Switzerland

Peter Fallesen, Sweden

Andrés Fandiño-Losada, Colombia

Xing Lin Feng, China

Tamas Ferenci, Hungary

Romulo Fernandes, Brazil

Emily Finne, Germany

Maddalena Fiordelli, Switzerland

Gilbert Fokou, Côte d’Ivoire

Mario Fordellone, Italy

Eduardo Fuentes-López, Chile

Rajendra Gadhavi, India

Andrea Madarasova Geckova, Slovakia

Raf Van Gestel, Netherlands

Laurent Getaz, Switzerland

Koustav Ghosh, India

Alessandro Gialluisi, Italy

Vincenza Gianfredi, Italy

Emilio Gianicolo, Germany

Ingrid Gilles, Switzerland

Annalisa Giosuè, Italy

Dominique Girard, Canada

Justin Glavis-Bloom, United States

Justyna Godos, Italy

Ana Rita Goes, Portugal

Pedro Goldberg, United States

Sergio Alejandro Gomez-Ochoa, Germany

Daniela Gonçalves-Bradley, United Kingdom

Patrick G. Goodman, Ireland

Vanessa Gorasso, Belgium

Reinhard Griebenow, Germany

Marlon Grodd, Germany

Yulong Gu, United States

Manuel Guedes, Portugal

Tee Guidotti, Canada

Elodie Guillaume, France

Gabriel Gulis, Denmark

Uwe Güth, Switzerland

Jennifer Guthrie, Canada

Meoïn Hagège, France

Bing Han, China

Larry Han, United States

Ziqiang Han, China

Sebastien Haneuse, United States

Iris Hartog, Netherlands

Mohammad Adrian Hasdianda, United States

Miriam Haverkamp, Germany

Fauna Herawati, Indonesia

Florian Herbolsheimer, Germany

Viviana Hernandez, Chile

Kimihiro Hino, Japan

Maren Hintermeier, Germany

Jan Hlodak, Slovakia

Gerard Hoek, Netherlands

Barbara Hoffmann, Germany

Hinke Hoffstädt, Netherlands

Anna-Clara Hollander, Sweden

Jian Hou, China

Guoqing Hu, China

Ravish Huchegowda, India

Joaquin Hurtado, Uruguay

Daniela Husarova, Slovakia

Aleksandra Ignjatovic, Serbia

Olutoyin Opeyemi Ikuteyijo, Switzerland

Wanrudee Isaranuwatchai, Thailand

Julien Issa, Poland

Natalja Jackmann, Sweden

Slobodan Jankovic, Serbia

Christian Gerd Janssen, Germany

Cláudia Raquel Jardim Santos, Portugal

Danijela Jerotijević, Slovakia

Enshe Jiang, China

Galia Jiménez, Spain

Anne Jolivet, France

Andrea Kaiser-Grolimund, Switzerland

Egide Kalisa, Canada

Hatem Kallel, French Guiana

Kalu Kalu, Zambia

Emir Karacaglar, Türkiye

Viktoryia Karchynskaya, Slovakia

Abhradeep Karmakar, United States

Sakari Karvonen, Finland

Natalia Kascakova, Czechia

Sarabpreet Kaur, India

Maryam Khoramrooz, Iran

Hugh Klein, United States

Victoria Klemm, Germany

Dorota Kleszczewska, Poland

Bojana Knežević, Croatia

Peter Koch, Germany

Christopher Kofahl, Germany

András Költő, Ireland

Łukasz Kominek, Poland

Jaroslava Kopcakova, Slovakia

Michaela Kosticova, Slovakia

Kpandji Isidore Kouadio, Côte d’Ivoire

Eleni Koutsogeorgou-Andreou, United Kingdom

Hana Knezevic Krajina, Croatia

Tyra Grove Krause, Denmark

Katarzyna Krot, Poland

Hanneke Kruize, Netherlands

Nino Kuenzli, Switzerland

Trésor Carsi Kuhangana, Democratic Republic of Congo

Kitti Kutrovatz, Hungary

Nikol Kvardová, Czechia

Emmanuel Lagarde, France

Pierre Laloux, Belgium

Agneta Larsson, Sweden

Miriam Laugesen, United States

Guy Launoy, France

Hyo Young Lee, Republic of Korea

Doo Woong Lee, United States

Lea Lenglart, France

Chu-Shiu Li, Taiwan

Pengzhu Li, Germany

Marta Lima-Serrano, Spain

Chien-Yu Lin, Taiwan

Elena Lisá, Slovakia

Silvia Lisciani, Italy

Keyang Liu, Japan

Laura Llambi, Uruguay

Giuseppina Lo Moro, Italy

Maria João Lobão, Portugal

Pablo Lopez, Argentina

Vincent Lorant, Belgium

Sofia Lotti, Italy

Mohamed Lounis, Algeria

Daniel Ludecke, Germany

Axel Luyten, Switzerland

Arno Maetens, Belgium

Javier Magaña Gómez, Mexico

Emanuella Gomes Maia, Brazil

Adriano Maia dos Santos, Brazil

Erma Manoncourt, France

Zhenxing Mao, China

Kailing Marcus, Switzerland

Magdalena Marczyńska, Poland

Claudia Martínez, Italy

Anneli Marttila, Sweden

Rainier D. Masa, United States

Milena Maule, Italy

Humphrey Mazigo, Tanzania

Joanna Teresa Mazur, Poland

Camilla Barbero Mazzucca, Italy

Edwin McIntosh, United Kingdom

David McQueen, Switzerland

Robert Mead, United States

Renald Meçani, Austria

Araya Medhanyie, Ethiopia

Roberto Mediavilla, Spain

Zdenek Meier, Czechia

Christos Melidis, France

Alexandra Mendoza-Graf, United States

Bruno Michel, France

Jose Mira, Spain

Hawri Mohammed-Ameen, Iraq

Michal Molcho, Ireland

Annie Montreuil, Canada

Leila Moradi, Iran

Mamothena Mothupi, United States

Shahzad Muhammad, Jordan

Guido Muharremi, Switzerland

Filip Mustač, Croatia

Joanna Myszkowska-Ryciak, Poland

Charlette Nangue, Cameroon

Rebecca Nantanda, Uganda

Antonia Napoletano, Italy

Sujita Narayan, Australia

Nawi Ng, Sweden

Qin Ng, Singapore

Nhung Nguyen, Vietnam

Thang Nguyen-Tien, Kenya

Will Nicholas, United States

Marie Nicolini, Belgium

Dorit Nitzan, Israel

Corinna Noel, United States

Elisabeth Nöhammer, Austria

Yves Jacket Nsavyimana, Burundi

Alexandre Nunes, Portugal

Jo-An Occhipinti, Australia

Sarah O'Connor, Canada

Issei Oi, Japan

Ijeoma Okedo-Alex, Nigeria

Joshua Okyere, Ghana

Juan Oliva-Moreno, Spain

Fernanda Oliveira, Brazil

Erin O’Loughlin, Canada

Jennifer Lee O'Loughlin, Canada

Stephen Okumu Ombere, Kenya

Michel Oris, Switzerland

Lyda Osorio, Colombia

Martina Otavova, Belgium

Anna Oudin, Sweden

Osaretin Oviasu, United Kingdom

Branwen Nia Owen, Switzerland

Veronika Pačutová, Netherlands

Jay Pan, China

Deb Prasad Pandey, Nepal

Salvatore Panico, Italy

Marcela Parada-Contzen, Chile

Roberta Parisi, Italy

Fabrizio Pasanisi, Italy

Eva Sophie Lunde Pedersen, Switzerland

Francisco Peinado, Spain

Melinda Pénzes, Hungary

Miguel Peralta, Portugal

Bilesha Perera, Sri Lanka

Julia Moreira Pescarini, United Kingdom

Gonzalo Piazzoni, Argentina

Nicola Pirastu, Italy

Thomas Plümper, Austria

Kaja Põlluste, Estonia

Ramesh Poluru, India

Lorenzo Postiglione, Italy

Paweł Prędkiewicz, Poland

Mauricio Quintero, Colombia

Ivana Radić, Serbia

Martina Ragettli, Switzerland

Alessia Raineri, Switzerland

Muthusamy Raman, India

Pilar Ramos, Spain

Harish Ranjani, India

Muhammad Adil Rasheed, Pakistan

Rashmi Rashmi, India

Shruta Rawat, India

Oliver Razum, Germany

Sophie Reale, United Kingdom

Alessio Rebechi, Luxembourg

Atta Ur Rehman, Pakistan

Bichen Ren, China

Marc Rendell, United States

Slađana Režić, Croatia

Ana Freitas Ribeiro, Brazil

Fulvio Ricceri, Italy

Woro Riyadina, Indonesia

Jaroslav Rosenberger, Slovakia

Magdalena Rosińska, Poland

Federica Rossetti, Belgium

Nazreen Rusli, Malaysia

Jihene Sahli, Tunisia

Anand Sajnani, Georgia

Giambattista Salinari, Italy

Diego San José, Spain

Alicia Sánchez-García, Spain

Omar Santangelo, Italy

T. Santhi Sri, India

Aurora Santin, Italy

Abdullah Sarıöz, Türkiye

Odile Sauzet, Germany

Carsten Schmidt, Germany

Lena Nilsson Schönnesson, Sweden

Lars Schwettmann, Germany

Aline Scohy, Belgium

Jamie Seabrook, Canada

Hana Sediva, United Kingdom

Tegbar Sendekie, Ethiopia

Maninder Setia, India

Anna Ševčíková, Czechia

Jonathan Shaffer, United States

Martine Shareck, Canada

Sukshma Sharma, Italy

Wegayehu Sheferaw, Australia

Victoria Shepherd, United Kingdom

Vladimir Shkolnikov, Germany

Krithiga Shridhat, India

Erik Sigmund, Czechia

Dagmar Sigmundová, Czechia

Luísa Silva, Brazil

Vittorio Simeon, Italy

Michael Simon, Switzerland

Titiksha Sirari, India

Amy Sitapati, United States

Ivana Skoumalova, Slovakia

Kastytis Šmigelskas, Lithuania

Emmanouil Smyrnakis, Greece

Kyung-Bok Son, Republic of Korea

Samriddhi Soni, United States

Sajid Bashir Soofi, Pakistan

Hannah Soong, Australia

Massimo Stafoggia, Italy

Christiane Stock, Germany

Sabrina Stöckli, Switzerland

Erwin Stolz, Austria

L. Suzanne Suggs, Switzerland

Dewi Susanna, Indonesia

Eric Joel Tahou, Côte d’Ivoire

Marcel Tanner, Switzerland

Ana Sofia Tavares, Portugal

Fabrizio Tediosi, Italy

Wudalew Tesfaye, United States

Tigabu Kidie Tesfie, Ethiopia

Kanokwan Tharawan, Thailand

George Thurston, United States

Sarina Till, South Africa

Brahmana Askandar Tjokroprawiro, Indonesia

Jason Torres, Spain

Eni Tresa, Albania

Milena Trifunovic-Koenig, Germany

Gudina Terefe Tucho, Ethiopia

Matthew Tuson, Australia

Izzatur Rahmi Mohd Ujang, Malaysia

Shin Ushiro, Japan

Sarah Uthoff, Germany

Maria Vaalavuo, Finland

Maija Vahteristo, Finland

Maja Vajagic, Croatia

Patricio Valenzuela, Chile

Laura Van Den Borre, Belgium

Pavla Vaneckova, Australia

Tuulikki Vehko, Finland

Lena Vergara, Colombia

Zuzana Veselská, Slovakia

Marcela Veytia-López, Mexico

Aleksandar Višnjić, Serbia

Matteo Vitali, Italy

Viktor Von Wyl, Switzerland

Isidora S. Vujcic, Serbia

Vesna Vujic-Aleksic, Bosnia and Herzegovina

Eliseu Waldman, Brazil

Jordyn Wallenborn, Switzerland

Samuel Wambugu, United States

Mkhululi Wami, Netherlands

Anqi Wang, United States

Siquan Wang, United States

Xuanxuan Wang, China

Grace Waweru, Kenya

Christian Wells, United States

Jason Were, Canada

Lawrence Wheat, United States

Frank Wieber, Germany

Albert W. Wu, United States

Rajesh Yadav, United States

Neşe Yakşi, Türkiye

Ferhat Yildiz, Türkiye

Mesut Yıldız, Türkiye

Hongzhao You, China

Ilknur Yuksel-Kaptanoglu, Türkiye

Ryszard Zaba, Poland

Iris Zachary, United States

Norhasmah Mohd Zain, Malaysia

Adam Zaludek, Czechia

Hamed Zandian, United Kingdom

Aurel Zelko, Slovakia

Andreja Brajsa Zganec, Croatia

Xing Zhao, China

Frederick Zimmerman, United States

Luis Zingg, Netherlands

